# A World of Babies

**Published:** 1887-03-12

**Authors:** 


### A WORLD OF BABIES.

BY ONE OF THE CROWD.

An Eastern Pilgrimage.

STEPNEY CAUSEWAY, even at the best of times, is not a very inviting thoroughfare, but approached through the pouring rain, on a cold, miserable day just before Christmas, the neighbourhood impresses one with its utter desolateness and chronic poverty. Commercial Road, its big shops decked in honour of the festive season with an eye only to striking effect, is, it is true, quite close, but even that broad highway fails to redeem the too obvious hard-upishness of the streets branching out of it. But my concern is not with the East End generally, but with the East End babies, and particularly with that representative fraction of them who use that infantile hotel and boarding-house known as Mrs. Hilton's *Crèche*. The first person we meet in Stepney Causeway is a boy in Dr. Barnardo's well-known uniform; and, in reply to our inquiry, he tells us promptly and politely the whereabouts of the place we are seeking. Not that much search was needed. The three ten-roomed houses which contain the *Crèche*, Home, and Infirmary, have conspicuous notices on the doors and windows for the guidance of strangers and visitors. A ring at the bell of No. 16 brings down a sharp little nurse-girl, who, as soon as she hears our business, invites us into a dark little parlour, and goes in quest of the matron. Even if we had not known the fact before, a glance at this room would convince us of two things—first, that the Home has not a farthing more of funds than it absolutely needs; and secondly, that not a penny more is spent than can be avoided on anything but the needs of the little inmates. Indeed, this room—Mrs. Hilton's own room, I believe, it is called—can only be described as shabby; it is crowded, too, with articles of every description, and in one corner was a great wooden box, evidently but just arrived, on the address label of which I read—"*Truth* Toy Fund." On one table there are portraits of some of the children who have been in the Home; hanging against the wall is a letter from Princess Christian, who takes a warm interest in this, as in so many other charitable works; and on another table is a box for contributions, with a visitors' book lying beside it. A glance at this latter shows how world-wide is the notoriety which Mrs. Hilton's good work has obtained, for it contains the names of visitors from America, the Colonies, and most of the countries of Europe. On this table also are copies of Mrs. Hilton's fifteenth "*Crèche* Annual," from which little book I learn how straitened are the means of this most excellent institution, and how enormous is the good it does, in a quiet way, in this crowded and poverty-stricken East End.

Precautions.

By this time the matron has arrived, and without any more ado we are shown over the establishment, first climbing up to the topmost storey of the house. As we ascend the steep winding stairs—for the houses are ancient—we catch a glimpse of a large and well-arranged playground, where, in more genial weather, the little inmates of the Home take recreation and exercise. We notice, too, that from the skylight on the roof to the basement a sort of well has been formed, through which a strong current of air is passing, and that on the wall are hung a number of bags. These, we are told, contain the clothes of the children brought in that morning, and we afterwards discover that each baby on its arrival is stripped, washed, and dressed in neat blue and white cotton garments; its own clothes being replaced, after another washing of the little body, when it is taken away at six o'clock in the evening. Each infant has its own clean towel, brush, etc., so that no risk of ophthalmic or other contagion can arise.

Babies, babies everywhere.

But it is time I said something about the *Crèche* itself. Arrived at the top of the stairs, we come at once to the nursery, a large, spotlessly clean, and most cheerful apartment. Cosy little cots are ranged round the wall, some of which are occupied; women are seated near the fire nursing, with evident care, a child on either knee; and, in the middle of the room, several children are tumbling about in great glee upon a large mattress, round which is placed a cage like arrangement of iron bars. These urchins are just old enough to crawl and tumble, so they are put here out of harm's way. All the children in this room, and in some others, belong to women who have to leave home to go to work, some to factories, others as washerwomen, charwomen, and the like. A goodly proportion of these poor people are widows, or have been deserted by their husbands, but not a few of the mothers have to "turn out" because he who should be the bread-winner can find nothing to do. "One hears constantly that trade is improving," said the matron ; "but we see no signs of it here." There are some very young children in this room, a proof of the stern necessity which drives the mother, often before she is at all able, again to the scouring-brush, the washtub, or the factory. Only a week before our visit, one of the inmates of this upper room was a baby only a fortnight old. Although a few of these infants are nice-looking enough, the majority are far from well-favoured ; they have been born, many of them, with the stamp of misery upon their faces, and it needs no one to tell me that the women whose energies have kept alive this institution through so many years, must have thoroughly imbibed the spirit of the Master's injunction. It is easy enough to grow enthusiastic over a chubby, well-fed crowing specimen of babyhood, whose merry laugh and dimpled limbs testify to the constant care of mother and nurse ; but it is quite another thing to attend to these children, who come in at uncertain intervals, whose flesh is pale and flabby, whose faces seldom wear a smile, and more often have upon them the shadow of the coming life-troubles, and whose eyes have none of the happy sparkle of the baby of well-to-do parents.

Parlour Boarders.

But what a contrast there is between these promiscuous inmates of the *Crèche* and the Infirmary (in which the sick children are nursed whilst their mothers are away) and the permanent occupants of Mrs. Hilton's establishment. The elder children of the first-mentioned class receive the visitor with a quiet stare, and very soon resume their picture-books or their toys ; those in the Home come up to you with a smile and are friends at once. They are healthy, well-cared for, and happy ; but the *Crèche* children, however affectionate their mothers may be, cannot possibly escape from the evils which have been their environment since birth—over-crowding, unsanitary arrangements, insufficient or improper food —as soon as they leave the friendly roof in Stepney Causeway. Some very pathetic stories are connected with the children in the Home, but for these I would refer the reader to Mrs. Hilton's *Annual* before-mentioned, which is sold for a nominal sum. It will be sufficient here to state that this Home is intended for the reception of children whose parents are dead or whose mothers are compelled for a shorter or longer period to leave them —sometimes to stay in a Convalescent Home, sometimes (as in the case of widows) to allow them to enter domestic service, and so on. Occasionally, the children are kept at the Home long enough to be trained for service themselves, and of course several of the inmates are over five years of age. These are taught in the schoolroom of the Home, and, judging from the way they sang a song for us, and from our inspection of their marking, knitting, etc., they would seem to be quite as advanced as scholars of their age in any neighbouring Board School. In addition to all this, Mrs. Hilton has a country-house at Feltham where the children are sent for change of air, and the wearied nurses for relief and recreation.

Some Figures.

Now for a very few statistics, which will tell, in the most powerful fashion, what the institution has done and is doing. It is in its sixteenth year of active operation. In the *Crèche* and Infirmary, from 80 to 100 children per day are received—the number last year being 30,240—and are washed, fed, clothed, and tended for 2*d.* per day of twelve hours—or less than 2*d.* when the circumstances of the mother are very bad. In the Orphan Home there are forty residents from four to ten years of age, at which latter period they are, if not claimed before by their friends, sent to some other institution ; and at Feltham there is accommodation for twenty-four young ones, whose ages may range from six months to four years. The value of the Home may be imagined when I add that last year there were 200 written and 160 personal applications, which have been classified as follows :—Widows with 67 children to provide for ; widowers with 48 ; men deserted by their wives, 14 ; women deserted by their husbands, with 30, and so on.

Hard up.

Will it be believed that all this good is being done for the most helpless and the most innocent of the whole human family, literally on a hand-to-mouth principle ? Always near the end of its resources, no department of the work has ever had to be closed. Somehow or other, help has always come at the last moment. But commercial depression is acting with twofold force to increase the care and trouble of the *Crèche's* founder and her friends—it has stopped some of the subscriptions, and increased the work which the institution must perform amongst the destitute children—and now there is serious talk about closing the Country Branch if further funds are not available this spring. I will add but one other word. Mrs. Hilton has arranged to hold a bazaar next May in aid of the *Crèche's* empty treasury.

